# Maternal-foetal transfer of *Plasmodium falciparum* and *Plasmodium vivax* antibodies in a low transmission setting

**DOI:** 10.1038/srep20859

**Published:** 2016-02-10

**Authors:** Sarah C. Charnaud, Rose McGready, Asha Herten-Crabb, Rosanna Powell, Andrew Guy, Christine Langer, Jack S. Richards, Paul R. Gilson, Kesinee Chotivanich, Takafumi Tsuboi, David L. Narum, Mupawjay Pimanpanarak, Julie A. Simpson, James G. Beeson, François Nosten, Freya J. I. Fowkes

**Affiliations:** 1 Macfarlane Burnet Institute of Medical Research, Melbourne, Australia; 2 Shoklo Malaria Research Unit, Mahidol-Oxford Tropical Medicine Research Unit, Faculty of Tropical Medicine, Mahidol University, Mae Sot, Thailand; 3 Mahidol-Oxford Tropical Medicine Research Unit, Faculty of Tropical Medicine, Mahidol University, Bangkok, Thailand; 4 Centre for Tropical Medicine and Global Health, Nuffield Department of Medicine, University of Oxford, Oxford, United Kingdom; 5 Department of Medicine, University of Melbourne, Australia; 6 Department of Immunology, Monash University, Alfred Medical Research and Education Precinct, Melbourne, Australia; 7 Department of Clinical Tropical Medicine, Faculty of Tropical Medicine, Mahidol University, Thailand; 8 Division of Malaria Research, Proteo-Science Center, Ehime University, Matsuyama, Japan; 9 Laboratory of Malaria Immunology and Vaccinology, NIAID/NIH, Rockville, MD, USA; 10 Centre for Epidemiology and Biostatistics, University of Melbourne, Australia; 11 Department of Epidemiology and Preventive Medicine, Monash University, Melbourne, Australia; 12 Department of Infectious Diseases, Monash University, Melbourne, Australia

## Abstract

During pregnancy immunolglobulin G (IgG) antibodies are transferred from mother to neonate across the placenta. Studies in high transmission areas have shown transfer of *P. falciparum*-specific IgG, but the extent and factors influencing maternal-foetal transfer in low transmission areas co-endemic for both *P. falciparum* and *P. vivax* are unknown. Pregnant women were screened weekly for *Plasmodium* infection. Mother-neonate paired serum samples at delivery were tested for IgG to antigens from *P. falciparum*, *P. vivax* and other infectious diseases. Antibodies to malarial and non-malarial antigens were highly correlated between maternal and neonatal samples (median [range] spearman ρ = 0.78 [0.57–0.93]), although *Plasmodium* spp. antibodies tended to be lower in neonates than mothers. Estimated gestational age at last *P. falciparum* infection, but not *P. vivax* infection, was positively associated with antibody levels in the neonate (*P. falciparum* merozoite, spearman ρ median [range] 0.42 [0.33–0.66], *Pf*VAR2CSA 0.69; *P. vivax* ρ = 0.19 [0.09–0.3]). Maternal-foetal transfer of anti-malarial IgG to *Plasmodium* spp. antigens occurs in low transmission settings. *P. vivax* IgG acquisition is not associated with recent exposure unlike *P. falciparum* IgG, suggesting a difference in acquisition of antibodies. IgG transfer is greatest in the final weeks of pregnancy which has implications for the timing of future malaria vaccination strategies in pregnant women.

The blood stage of *Plasmodium falciparum* and *P. vivax* malaria infections are a major cause of mortality and morbidity, resulting in an estimated 584,000 deaths and 198 million clinical cases each year, predominantly young children under the age of five^1^. Despite these increased risks early in childhood, clinical malaria in the first six months of life is generally uncommon and infections tend to be asymptomatic with low density parasitaemia^2^. This protection in infancy is often attributed partially to the passive transfer of naturally acquired protective immunity to malaria from mother to child prior to the development of the infant’s own immune system^2^,^3^.

Naturally acquired immunity develops in individuals living in malaria endemic areas after repeated exposure to *Plasmodium* spp. infections. Immunity acts by reducing parasite densities and associated clinical symptoms rather than protecting against *Plasmodium* spp. infection *per se*^4^. Antibodies are an important component of malarial immunity^5^,^6^ and targets include antigens on the surface of sporozoites (pre-erythrocytic stage), merozoites (involved in erythrocyte invasion) and antigens on the surface of infected erythrocytes (IEs)^1^,^6^,^7^,^8^. Antibodies to the blood stage targets are associated with protection against clinical disease^2^,^7^,^9^,^10^ and individuals with substantial immunity are likely to possess a large repertoire of antibody responses^2^,^3^,^11^. At the time of their first pregnancy, women living in malaria endemic areas will have developed a degree of protective immunity. Despite this pre-existing immunity pregnant women typically develop higher *P. falciparum* and *P. vivax* densities, compared to non-pregnant adults^4^,^12^. This susceptibility has been attributed to immune modulation resulting in an impaired ability to limit parasite replication during pregnancy, and the lack of immunity to placental-binding variants of *P. falciparum* that accumulate in the placenta^5^,^6^,^13^. The sequestration of *P. falciparum*-IEs in the placenta is mediated in part by *Pf*VAR2CSA, a specific variant of *P. falciparum* erythrocyte membrane protein (*Pf*EMP1) expressed on the surface of *P. falciparum*-IEs^14^. Over successive pregnancies, women resident in malaria-endemic areas in Africa and Asia acquire *Pf*VAR2CSA antibodies which have been associated with a decrease in the rates and density of placental infection^13^,^15^.

Although foetuses are capable of synthesising immunoglobulin from the twelfth week of gestation, the majority of foetal immunoglobulin is of maternal origin^16^,^17^. The maternal-foetal transfer of immunoglobulins begins in the sixteenth week of gestation^16^ and requires active transport via Fc receptors on placental syncytiotrophoblasts^18^. The immunoglobulin G (IgG) isotype is the only immunoglobulin to cross the placenta in significant amounts, facilitated by the neonatal Fc receptor^18^,^19^. Anti-*P. falciparum* IgG has been shown to correlate between maternal and cord samples, and detectable IgG titres and *P. falciparum* antigen-specific antibodies have been demonstrated in newborns living in high transmission areas of Africa and Papua New Guinea^20^,^21^,^22^,^23^,^24^. There is a paucity of maternal-foetal transfer studies of *P. falciparum* in low transmission settings and even fewer studies addressing the transfer of *P. vivax* antibodies. Importantly there are few studies comparing the maternal-foetal transfer of antibodies to *Plasmodium* spp. compared to other pathogens and vaccine-preventable diseases. In addition, very little is known about factors that influence infant antibody levels and, importantly, that influence the rate of maternal-foetal antibody transfer. Previous studies have shown that *P. falciparum* placental infection, HIV, gestational age at birth and hypergammaglobulinemia can reduce transplacental transfer of maternal antibodies^25^,^26^,^27^,^28^, but other factors may also play a role.

In this study we determined antibodies to a panel of *P. falciparum* and *P. vivax* antigens representing different life-cycle stages in maternal, umbilical cord, and neonatal samples at delivery, in Karen women attending antenatal clinics at the Thai-Myanmar border. In this setting both *P. falciparum* and *P. vivax* transmission is low and placental infection is relatively rare as is the presence of HIV (<0.2%)^29^. We investigated maternal-foetal transfer of antibodies towards sporozoites, *P. falciparum* and *P. vivax* merozoite antigens, and antigens on the surface of *Pf*-IEs and gametocytes as well as to tetanus, measles and cytomegalovirus (CMV). We investigated how factors such as species-specific *Plasmodium* exposure (and timing of exposure), gravidity, chemoprophylaxis and gestational age influenced maternal-foetal transfer and neonatal antibody levels.

## Materials and Methods

### Study population

This study took place in the antenatal clinics (ANCs) of the Shoklo Malaria Research Unit (SMRU) in north-west Thailand from November 1998 to January 2000. More than 90% of pregnant women in the camps attended SMRU ANCs on a weekly basis^30^. All women are invited to come to an ANC as soon as they are aware of their pregnancy. All women who attend ANCs are screened weekly for *Plasmodium* spp. infection by light microscopy using a finger prick blood sample, and every second week for anaemia by haematocrit. All women are invited to deliver at SMRU although Karen women traditionally deliver at home. The epidemiology of malaria in this area, and the effects of *P. falciparum* and *P. vivax* malaria during pregnancy and on birth outcomes, have been described in detail previously^30^,^31^,^32^.

### Study design and data collection

Mother-neonate pairs at delivery were selected from women included in a case-control study of *Plasmodium* spp. immunity, nested in a placebo-controlled trial of chloroquine prophylaxis^33^,^34^. Briefly, four tablets of chloroquine (153 mg base) or placebo were given at enrolment, and 2 tablets of the same type on a weekly basis until delivery. For more details on treatment refer to Villegas *et al.*, 2007^34^. Plasma samples at delivery were taken from peripheral samples of the mother and neonate (heel prick) as well as cord samples. Samples were available for 57/136 cases (women with *Plasmodium* spp. parasitaemia detected by light microscopy at any time during pregnancy) and 111/331 controls (women with no parasitaemia at any time during pregnancy). The controls represented a subset, selected on IgG response to schizont extract at enrolment for determination of antibody responses during pregnancy^33^. These were 74 of the most reactive individuals (‘high responder controls’) together with 37 randomly selected sero-negative individuals (see flow chart Supplemental Fig. 1). Apart from the selected subset, unavailable samples are the result of sample not being taken as the women delivered at home, or insufficient sample volume available for antibody determination. Estimated gestational age (EGA) was primarily assessed by the Dubowitz method^35^ and, when possible, neonates had a newborn neurological examination^36^. When a Dubowitz evaluation could not be done, EGA was calculated by a formula developed from a cohort of Karen pregnant women with known gestation age [(fundal height on admission ×0.887) + 4.968 weeks]^31^ which performs similarly to the Dubowitz method in this population^37^. A premature birth was defined as delivery before 37.0 estimated weeks of pregnancy.

Written informed consent was obtained from all participants. The study was performed in accordance with the guidelines approved by the Ethics Committee of the Faculty of Tropical Medicine of Mahidol University, Thailand; the Ethics Committee of the London School of Hygiene & Tropical Medicine, UK; and the Walter and Eliza Hall Institute of Medical Research, and the Alfred Hospital, Australia.

### Antibody determination

Total IgG was determined to antigens representing a range of life-cycle stages from both *P. falciparum* and *P. vivax* that represented both biomarkers of exposure and protective immunity^9^,^10^. *P. falciparum* (3D7 allele unless otherwise stated) merozoite antigens apical membrane antigen 1, (*Pf*AMA1), erythrocyte binding antigen 175 (*Pf*EBA175) regions III-V, merozoite surface protein (MSP) 2 (*Pf*MSP2), *Pf*MSP3; infected erythrocyte antigen *Pf*VAR2CSA (Duffy binding like (DBL) 5ε domain as it is recognized across geographically diverse isolates compared to other domains which are highly strain-specific^38^, 7G8 allelle), and *P. vivax* merozoite antigen (*Pv*AMA1) was measured by high-throughput ELISA as previously described^33^.

Total IgG was also determined to *P. falciparum* merozoite antigens EBA 140 regions II and III-V (*Pf*EBA140-RII and *Pf*EBA140-RIII-V)^39^, reticulocyte binding protein homologue 2 (*Pf*Rh2)^40^ and *P. vivax* merozoite antigens *Pv*MSP1_19_^41^, and Duffy binding protein (*Pv*DBP)^42^, *P. falciparum*-IE surface antigen *Pf*DBLα (IT4var14 NTSB3-DBLa00.23)^43^ and the *P. falciparum* sporozoite protein (*Pf*CSP)^44^ and gametocyte antigens (*Pf*s230)^45^, and antigens to tetanus toxoid, cytomegalovirus (CMV) and measles (PROSPECbio) using a Janus robotic platform but otherwise performed as described previously^33^. Antigens were coated on plates at 0.5 μg/mL and human sera were tested to each antigen at the following dilutions; *Pf*DBLα, *Pf*DBL5ε, *Pf*s230, *Pv*AMA1 and measles at 1:250, *Pf*MSP2, *Pf*MSP3, *Pf*EBA140-RII, *Pf*EBA140-RIII-V, *Pf*EBA175*, Pf*Rh2, *Pf*CSP, tetanus toxoid and CMV at 1:500, *Pv*MSP1_19_ and *Pv*DBP at 1:1000 and *Pf*AMA1 at 1:2000 (Supplemental Table 1). Seropositivity was defined as an optical density (OD) greater than the mean plus three standard deviations of 17 negative controls (non-exposed Melbourne donors).

### Statistical Analysis

Spearman’s rank correlation coefficients (ρ) were calculated to measure the association between antibody levels derived from maternal, cord and neonatal blood, and for those mothers with a malaria infection during pregnancy, between antibody levels and estimated gestational age of last exposure to malaria infection. To investigate potential predictors of neonatal antibody levels we used multivariable robust linear regression (to reduce the influence of multivariate model outliers) on log_e_ transformed antibody levels. Variables of interest included maternal antibody levels (log_e_ transformed), gravidity (primigravid, multigravida), chloroquine prophylaxis (chloroquine, placebo) and EGA at delivery in weeks (continuous). In addition a new variable was created from case-control status to represent species-specific exposure. We defined an “exposed case” as a woman who had been exposed during pregnancy to the species representing the antigen, so that *P. falciparum* antibodies could be related to *P. falciparum* exposure and *P. vivax* antibodies to *P. vivax* exposure. Each species-specific case may also have had infection with the other parasite species. In addition controls were divided into high and low responders which were selection criteria for inclusion in the immunological study^33^. Variables were investigated in two separate multivariable models for each antigen. The first model excluded maternal IgG levels at delivery to investigate whether *Plasmodium* spp. exposure, gestational age at delivery, gravidity or chloroquine prophylaxis are associated (either directly or indirectly via maternal IgG levels at delivery) with neonatal IgG levels. The second model included maternal IgG levels to investigate whether variables of interest were associated with transfer of maternal antibodies to the foetus. To investigate whether gestational age at delivery, gravidity or chloroquine prophylaxis modified the transfer of maternal antibodies to the foetus, an interaction term with maternal IgG levels and estimated gestational age (<40 weeks, 40–41 weeks, >41 weeks), or gravidity, or chloroquine prophylaxis were investigated. These multivariate analyses were also conducted on the ratio of neonatal/maternal antibodies in seropositive women. Sensitivity analysis was performed by running the model including and excluding the 7 preterm deliveries in the <40 weeks category. Coefficients were less than 10% different between each model therefore preterm deliveries data were included in the <40 weeks group rather than a separate category containing few observations. Final models included maternal IgG levels together with an interaction term with estimated gestational age with adjustments for gravidity, chloroquine prophylaxis and *Plasmodium* spp. exposure. Analyses were conducted with Stata version 13.0 (Stata Corporation, College Station, Texas, US).

## Results

Maternal characteristics and birth outcomes of the 57 cases and 111 controls included in the current study are shown in Table 1. Briefly, the majority of women were multigravida (77.2% cases, 85.6% controls) and the median estimated gestational age at delivery was 40 weeks. In cases, 63.2% of women experienced a *P. falciparum* infection, and 57.9% experienced a *P. vivax* infection during pregnancy, the median number of infections for both species was one (Table 1).

**Figure 1:**
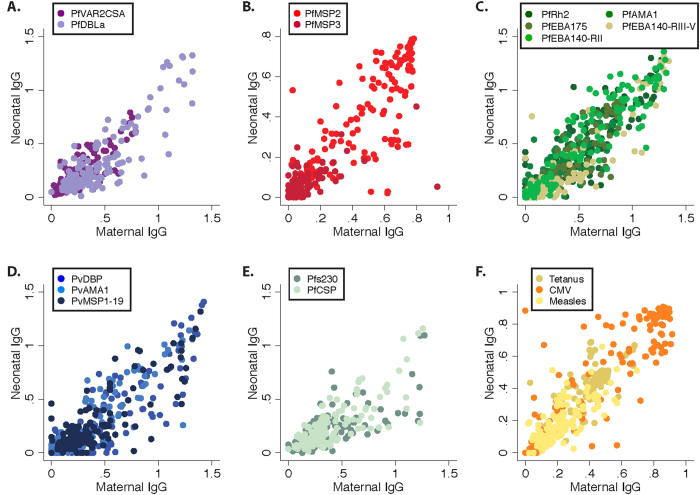
Scatterplots of maternal and neonatal malarial antibody levels. Scatterplots of maternal and neonatal antibody levels according to antigens representing (**A**) the surface of *P. falciparum* infected erythrocyte (*Pf*EMP1 domains), (**B**) *P. falciparum* merozoite surface, (**C**) *P. falciparum* merozoite invasion ligands, (**D**) *P. vivax* merozoite, (**E**) *P. falciparum* sporozoite and gametocyte antigens, (**F**) other infectious diseases. *Plasmodium* spp. antibody levels, and antibodies to non-malarial antigens, were highly correlated between maternal and neonatal samples (median [range] spearman ρ = 0.78 [0.57–0.93]). Specific spearman ρ correlation results between maternal and neonate samples: *Pf*VAR2CSA 0.77; *Pf*DBLa 0.74; *Pf*MSP2 0.78; *Pf*MSP3 0.57; *Pf*Rh2 0.84; *Pf*EBA175 0.91; *Pf*EBA140-RII 0.93; *Pf*EBA140-RIII-V 0.83; *Pf*AMA1 0.84; *Pv*DBP 0.78; *Pv*AMA1 0.87; *Pv*MSP1-19 0.68; *Pfs*230 0.75; *Pf*CSP 0.75; Tetanus 0.67; CMV 0.84; Measles 0.75.

**Figure 2:**
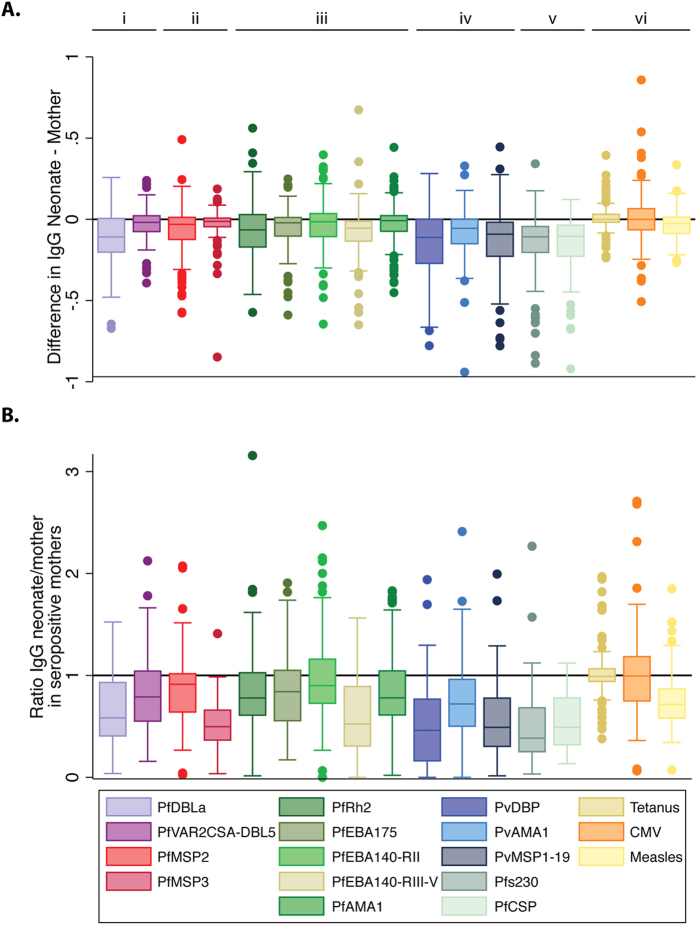
Difference in total IgG and ratio of total IgG between maternal and neonatal samples in seropositive women. Box plot (median, interquartile range, range) of (**A**) the difference in total IgG between maternal and neonatal samples (mother-neonate), and (**B**) the ratio of IgG in neonate/mother samples in order of antigens representing i) *P. falciparum* infected erythrocyte, ii) *P. falciparum* merozoite surface, iii) *P. falciparum* merozoite apical (microneme and rhoptry), iv) *P. vivax* merozoite, v) *P. falciparum* transmission antigens, vi) infectious diseases. Line indicates no difference between maternal and neonatal IgG. There tends to be less neonatal IgG than maternal IgG to malaria antigens, which is not observed for CMV or Tetanus toxin antigens.

Antibody levels were determined towards *P. falciparum* and *P. vivax* antigens in maternal, cord and neonatal samples (maternal seroprevalences are summarised in Table 1). In addition IgG to non-malarial antigens tetanus toxoid, CMV and measles were also determined. Both *Plasmodium* spp. antibody levels, and antibodies to non-malarial antigens, were highly correlated between maternal samples and cord (median [range] spearman ρ = 0.89 [0.68–0.94]) and neonatal samples (0.78 [0.57–0.93], Fig. 1). The absolute difference in antibody levels in mother and neonate samples was calculated within each mother-neonate pair and showed that neonate IgG levels were typically lower for *Plasmodium* spp. antigens, but for each antigen there was variation in the amount that was transferred to the neonate (Fig. 2A). Antibodies specific for measles were also lower in neonates, whereas levels of tetanus and CMV antibodies were comparable in maternal and neonate samples (Fig. 2). Analysis of maternal-foetal antibody ratios in seropositive women yielded similar findings (Fig. 2B).

### Species-specific infection and antibody transfer

To determine whether species-specific exposure during pregnancy resulted in species-specific antibody transfer we analysed the data by species of infection. Maternal *Plasmodium* species-specific exposure was associated with species-specific neonatal antibody levels, with higher neonatal *P. falciparum* antibody levels observed for those neonates whose mother experienced a *P. falciparum* episode in pregnancy (representative examples, Table 2). Similar associations were observed for *P. vivax* antibody levels in response to *P. vivax* infection, but were of lower magnitude than that observed for *P. falciparum* and only reached significance for *Pv*AMA1 (Table 2). No associations with neonatal antibody levels were observed for gravidity, chloroquine prophylaxis and EGA at delivery, in line with our previous analyses of antibody responses in pregnancy^33^.

Due to the weekly parasitological assessment, we were able to assess how the timing of infections during pregnancy influenced the levels of neonatal antibodies and maternal transfer of antibodies in a subset of women who had been exposed to *P. falciparum* (n = 36) and *P. vivax* (n = 33). We examined the correlation of antibody levels in the neonate at delivery with estimated gestational age of the last exposure (*P. falciparum* - median [range] 23.7 weeks [7.6–40.9], *P. vivax* - 27.1 [12.2–40.3]). EGA at last *P. falciparum* infection was positively associated with antibody levels in the neonate, with higher levels seen in neonates whose mothers had been exposed later on in pregnancy (*P. falciparum* merozoite, spearman ρ median [range] 0.42 [0.33–0.66], *Pf*VAR2CSA 0.69). There was no association between *P. vivax* antibody responses and EGA at the last *P. vivax* infection (ρ 0.19 [0.09–0.3]). There was no correlation between the time since maternal tetanus vaccination and neonatal tetanus antibodies at delivery (ρ −0.08).

### Maternal-foetal transfer effect modifiers

To identify factors that modified the maternal-foetal transfer of antibody levels, log transformed maternal antibody levels, together with interaction terms between variables of interest, were added to the model predicting neonate antibody levels. Gravidity and chloroquine prophylaxis were not shown to modify the relationship between maternal and neonatal antibody levels (data not shown), but EGA was an effect modifier of the maternal-foetal transfer relationship. The increase in neonatal antibody levels per unit increase in maternal antibody levels were typically moderately greater in neonates born after 40 weeks gestation compared to neonates born before 40 weeks gestation, with varying levels of magnitude of this effect depending on antigen (Table 3). Analysis of maternal-foetal antibody ratios in seropositive women also demonstrated, increasing maternal-foetal transfer with increasing EGA (Supplemental Table 2).

## Discussion

In a comprehensive study of the maternal-foetal transfer of *P. falciparum* and *P. vivax* antibody responses in an endemic low transmission area of South-East Asia, we demonstrated transfer of a broad range of both *P. falciparum* and *P. vivax* antibodies across the placenta during pregnancy. Anti-malarial IgG was slightly lower in neonates compared to mothers, but reached higher levels when gestation was longer. *P. falciparum* specific IgG was positively associated with recent *P. falciparum* infection, however *P. vivax* IgG was not as strongly linked with infection.

Antimalarial IgG levels were lower in neonates compared to mothers, a finding which has been reported in other malaria studies in high transmission areas^21^,^24^,^46^,^47^,^48^. Lower levels in neonates are potentially due to the saturation of neonatal Fc receptors which mediate the transfer of IgG across the syncytiotrophoblast^3^. Saturation of Fc receptors may be caused by maternal hypergammaglobulinemia (IgG > 15 g/L)^3^ which has been associated with reduced levels of anti-measles and anti-viral, but not tetanus, antibodies in the cord blood compared to the mother in several studies^27^,^28^,^49^,^50^. Similar findings were also observed in our study with lower level of anti-measles IgG observed in the neonate compared to the mother, but no differences in levels of anti-tetanus toxoid or anti-CMV. It is unclear what underpins differences in transfer according to antigen but similar to anti-measles antibody, the transfer of *Plasmodium* spp. antibody also appears impaired. Despite this impairment, our data would suggest that even in low transmission areas with prompt treatment of malaria, maternal IgG to malarial antigens is high and antimalarial antibodies are transferred to neonates at maximum capacity. Maternal-foetal transfer was dependent on EGA at delivery, with more maternal antibody transfer occurring at later stages of pregnancy. In healthy pregnancies (both term and preterm), neonatal total IgG is directly related to length of gestation^3^,^18^,^51^ with the maternal-foetal ratio increasing in the last four to six weeks suggesting an increase in active IgG transport during this time^51^,^52^. Our data provides evidence that increased transfer of anti-malarial antibodies also occurs in the last four weeks of pregnancy despite antibody levels being lower in the neonate at delivery.

The only maternal factor associated with neonatal antibody levels was exposure to *Plasmodium* spp. during pregnancy. Increases in *P. falciparum*-specific neonatal antibody levels were strongly associated with *P. falciparum* maternal infection during pregnancy. However increases in *P. vivax* antibody levels, with respect to *P. vivax* maternal exposure, were of lower magnitude. We have previously demonstrated in these women that *P. falciparum* merozoite and pregnancy-specific responses are boosted with each successive *P. falciparum* infection in pregnancy but *P. vivax* responses are not boosted in response to *P. vivax* infection^33^. This apparent lack of boosting is most likely due to the lower parasite densities observed in *P. vivax* infections during pregnancy in this cohort which may not be detected with microscopy^33^ or the presence of sub-microscopic infections from liver stage relapse (occasionally detected by microscopy)^53^ which result in relatively constant antibody concentrations. The ability to mount an antibody response upon *Plasmodium* spp. exposure during pregnancy has a knock-on effect on neonatal antibody levels, but how differential species-specific antibody responses translates to differential species-specific risk in the first six months is yet to be determined.

The weekly sampling for the presence of parasites also enabled us to accurately classify when exposure to *Plasmodium* spp. occurred during pregnancy, and investigate the association between timing of infection and neonatal antibody levels as previous studies of infant antibodies have only examined parasitaemia at delivery^26^,^46^,^47^. We found that antibody levels were higher in neonates born to women who had been infected more recently (particularly in the last trimester). Conversely, there was no association between timing of maternal tetanus immunization and anti-tetanus antibodies in the neonate. This may be due to differences in antibody longevity between tetanus and *Plasmodium* spp. antibodies. We have previously shown that antibody half-life is shorter for *Plasmodium* spp. antibodies (1–2 years) compared to tetanus antibodies (10–12 years) in this cohort^33^,^51^,^52^,^54^. The implications of the aforementioned increase in maternal-foetal antibody transfer in later stages of pregnancy and short-lived responses are that foetuses of mothers that experience malaria exposure early on in pregnancy, particularly those before four months gestation when transfer begins^16^, may acquire lower levels of protective immunity compared to those with mothers infected in the latter stages of pregnancy. This finding suggests that if future malaria vaccine programs consider administering the vaccine to pregnant women then the third trimester may be the optimal time of vaccination to boost antibody levels in the infant.

Antibody transfer may be affected by drug regimens designed to protect women from malaria during pregnancy. Women included in the current study were participating in a randomised controlled trial of chloroquine prophylaxis^34^. Chloroquine has been proposed to be immunosuppressive^55^ but for our data no association between chloroquine and infant antibody levels or maternal-foetal transfer was observed which is in line with a previous study in Tanzania^46^. Chloroquine would most likely act indirectly on infant antibody levels by reducing exposure to *Plasmodium* spp. antigens. In the trial chloroquine prophylaxis successfully prevented *P. vivax*, but not *P. falciparum* infection during pregnancy^34^. Other studies of intermittent preventative treatment of malaria in pregnancy (IPTp) have shown that IPTp can reduce pregnancy-specific antibody levels in pregnant women living in malaria endemic areas of Africa^56^,^57^. A large reduction in antibody levels due to IPTp could result in lower antibody levels in the infant, however in our study it appears that transfer is at maximum capacity even in women who were not exposed to malaria during pregnancy. The impact of IPTp on malaria risk in infancy is unknown.

This study was designed to assess anti-malarial antibody transfer in women in a low transmission setting. These women are also quickly treated on the presence of *Plasmodium* in a blood smear. Rapid treatment upon diagnosis of *Plasmodium* spp. infection may mean that high parasitaemias and placental malaria are reached less often and consequently lower levels of antibody are observed in our study population than in both high transmission settings and populations in low transmission settings who do not receive intensive screening and treatment. In this setting there are low numbers of premature births (<37 weeks, n = 7), and low prevalence of placental malaria (histopathological placental parasite positivity 4.3% (3.2–5.6))^29^. It is possible that there are larger differences in maternal-foetal transfer in higher transmission areas where preterm birth and placental malaria are more frequent. It has also been shown that MSP1-42 and tetanus toxoid antigens can cross the placenta into the foetal circulation as immune complexes with IgG^58^,^59^. This raises the possibility that not all IgG is available for binding in an ELISA assay and we may have underestimated the level of maternal-foetal transfer. In this study we measured total IgG but the IgG subclass of transferred antibodies may also be important. Neonatal Fc receptors preferentially transport IgG1, followed by IgG3, and IgG2^3^. The predominant subclasses against *Plasmodium* spp. antigens are IgG1 and IgG3 that mediate reductions in parasitemia by a number of mechanisms (e.g. opsonic phagocytosis, growth/invasion inhibition), but different *Plasmodium* spp. antigens have different IgG1/IgG3 bias^60^. Only one study has investigated functional immunity and shown that maternally transferred antibodies (MSP1-19) have growth-inhibitory activity^61^ but other mechanisms also need to be investigated. Our finding that anti-*Pf*EMP1 antibodies are transferred may also suggest an ability for transferred IgG to protect the neonate from severe malaria potentially via parasite clearance or adherence inhibition^62^,^63^,^64^. Further studies of functional immunity and clinical protection are warranted to elucidate the role that maternally transferred antibodies have in protection from malaria in infancy.

The clinical relevance of the antimalarial antibodies detected in infants is debated with conflicting evidence on the protective effects of passively transferred *P. falciparum* antibodies in the first year of life^2^,^21^,^26^,^47^,^65^,^66^,^67^. There are many possible reasons for these conflicting findings, such as differences in study design, host and environmental factors and malaria transmission. Rates of placental malaria may also be important because the presence of placental malaria is associated with foetal priming to blood stage antigens^68^,^69^,^70^. Importantly it is currently unclear how long maternal antibodies persist in the infant. Most transplacentally transferred IgG has a half-life of 21 days and is more-or-less undetectable by six months of age^61^. In addition, species-specific differences in potential protection due to antibody transfer are unknown. Exposure to *P. falciparum* led to increased levels of *P. falciparum* antibodies in the infant, but the effect of *P. vivax* exposure on *P. vivax* antibody levels was not as marked. How this translates to the differential risk of *P. falciparum* and *P. vivax* during the first six months of life is unknown and further studies are warranted.

## Conclusions

In a low transmission area in Thailand, we demonstrated the effective maternal-foetal transfer of both *P. falciparum* and *P. vivax* antibodies in treated malaria episodes. Infant antibody levels were influenced by maternal exposure to infection, and timing of those exposures. Functional assays are needed to determine the validity of the passive immunity hypothesis that maternal transfer of antibodies leads to protection from disease in infants in the first months of life. If this is indeed the case this work indicates that gestational age is important for antibody transfer, and timing of vaccination during pregnancy would also be important. If protective immunity can be gained from the mother, studies to determine the length of time that infants remain protected will inform the optimal timing of vaccination for infants.

## Additional Information

**How to cite this article**: Charnaud, S. C. *et al.* Maternal-foetal transfer of *Plasmodium falciparum* and *Plasmodium vivax* antibodies in a low transmission setting. *Sci. Rep.* **6**, 20859; doi: 10.1038/srep20859 (2016).

## Supplementary Material

Supplementary Information

## Figures and Tables

**Table 1:** Summary of maternal and infant characteristics, and antibody prevalence in cases and controls.

Characteristic	Cases (n = 57)	Controls (n = 111)
*Plasmodium* spp. during pregnancy		
*P. falciparum* infection	36 (63.2)	–
Number of *P. falciparum* infections	1 {0–1}, 0–5	–
*P. vivax* infection	33 (57.9)	–
Number of *P. vivax* infections	1 {0–2}, 0–5	–
Age, years	25.6 [7.2], 15–40	27.17 [6.3], 15–42
Smoker	32 (56.1)	52 (46.8)
Gravidity	3 {2–4}, 1–10	3 {2–5}, 1–13
Primigravid	13 (22.8)	16 (14.4)
Chloroquine prophylaxis	36 (63.2)	56 (50.5)
Maternal body mass index, kg/m^2^	20.7 [2.1], 16.1–27.8	21.4 [2.5], 14.2–29.5
Sex of baby, Male	33 (57.9)	74 (66.7)
EGA at delivery, weeks	39.7 [1.7], 33.6–42.2	40.1 [1.3], 31.6–41.5
Maternal antibody prevalence		
*P. falciparum* merozoite^a^ (% range)	68.4–93.0	57.0–85.3
*P. vivax* merozoite^b^ (% range)	57.9–61.4	38.5–63.3
*P. falciparum* VAR2CSA	45 (79.0)	63 (57.8)
*P. falciparum* DBLα	47 (82.5)	87 (78.9)
*P. falciparum* CSP, Pfs230	27 (47.4), 29 (50.9)	36 (33.0), 40 (36.7)
CMV, Tetanus toxoid	42 (82.4), 50 (87.7)	65 (60.8), 94 (86.2)
Measles	17 (33.3)	37 (34.6)

Values are mean [standard deviation], range; or median {inter-quartile range}, range; or N (%) unless otherwise specified. Discrepancies in percentages for maternal antibodies are due to two missing values in the controls. There was no significant difference between cases and controls in characteristics, excluding maternal antibody prevalences. EGA – estimated gestational age. ^a^*Pf*AMA1, *Pf*EBA175, *Pf*EBA140_RII_, *Pf*EBA140_RIII-V_ *Pf*MSP2 and *Pf*Rh2. Excluding *Pf*MSP3 outlier 27.5% and 23.4% in cases and controls respectively. *Pf*MSP3 has been shown to have low reactivity. ^b^*Pv*AMA1, *Pv*MSP1_19_, and *Pv*DBP.

**Table 2:** Multivariable linear regression of association between maternal characteristics and neonatal antibody levels.

Variable	*P. falciparum*						*P. vivax*			
	*Pf*VAR2CSA		*Pf*AMA1		*Pf*EBA140RII		*Pv*AMA1		*Pv*DBP	
	Exp(b) (95% CI)	*P*	Exp(b) (95% CI)	*P*	Exp(b) (95% CI)	*P*	Exp(b) (95% CI)	*P*	Exp(b) (95% CI)	*P*
Case-control exposure group										
Low control	1.00		1.00		1.00		1.00		1.00	
High control	1.03 (0.97–1.09)	0.29	1.08 (0.99–1.16)	0.07	1.24 (1.03–1.49)	**0.02**	1.10 (1.00–1.22)	0.06	1.01 (0.92–1.11)	0.87
Non-exposed case	0.98 (0.90–1.06)	0.58	0.98 (0.87–1.10)	0.69	0.86 (0.65–1.13)	0.28	1.23 (1.07–1.42)	**0.01**	0.99 (0.86–1.13)	0.87
Exposed case	1.16 (1.08–1.25)	**<0.001**	1.27 (1.15–1.40)	**<0.001**	1.48 (1.18–1.86)	**<0.001**	1.13 (0.99–1.29)	0.07	1.04 (0.92–1.18)	0.55
Multigravida	1.04 (0.98–1.11)	0.22	0.97 (0.89–1.05)	0.44	0.99 (0.81–1.21)	0.94	0.98 (0.88–1.10)	0.78	0.90 (0.81–1.00)	0.05
Chloroquine	0.99 (0.96–1.06)	0.67	1.03 (0.91–1.03)	0.34	1.08 (0.80–1.08)	0.34	0.96 (0.96–1.13)	0.32	1.02 (0.91–1.06)	0.66
EGA, weeks	1.00 (0.99–1.02)	0.82	1.02 (1.00–1.04)	0.12	1.03 (0.98–1.09)	0.25	1.02 (0.99–1.05)	0.2	1.00 (0.97–1.03)	0.97

Neonate antibody levels were log_e_ transformed for analysis and Exp(b) is the exponential of the model coefficients and represents the ratio of the geometric means of neonatal antibody levels. Comparison groups: Case-control exposure group, versus low responder controls; Multigravida, versus primigravida, chloroquine versus placebo, EGA – estimated gestational age is a continuous variable in weeks. Representative examples of antibodies to *Pf*-IE, *P. falciparum* and *P. vivax* merozoites are shown. Similar patterns were observed with other antigens (data not shown).

**Table 3:** Multivariable linear regression of the effect modification of estimated gestational age on the association between maternal and neonatal antibody levels.

EGA (weeks)	*P. falciparum*						*P. vivax*			
	*Pf*VAR2CSA		*Pf*AMA1		*Pf*EBA140RII		*Pv*AMA1		*Pv*DBP	
	Exp(b) (95% CI)	P	Exp(b) (95% CI)	P	Exp(b) (95% CI)	P	Exp(b) (95% CI)	P	Exp(b) (95% CI)	P
<40	1.95 (1.70–2.22)	Ref.	2.23 (1.99–2.49)	Ref.	2.44 (2.26–2.65)	Ref.	2.31 (2.11–2.52)	Ref.	1.99 (1.75–2.25)	Ref.
40–41	2.44 (2.16–2.75)	0.008	2.03 (1.83–2.24)	0.2	2.48 (2.30–2.68)	0.77	2.30 (2.10–2.53)	0.99	1.90 (1.71-2.11)	0.58
>41	2.08 (1.72–2.51)	0.57	2.69 (2.30–3.15)	0.047	2.66 (2.37–2.98)	0.22	2.67 (2.29–3.11)	0.1	2.61 (2.30–2.96)	0.003

Neonatal and maternal antibody levels were log_e_ transformed for analysis and Exp(b) is the exponential of the model coefficients and represents the ratio of geometric means of neonatal antibody levels. Correlations between maternal and neonatal antibody levels were statistically significant for all EGA categories (P < 0.001). Representative examples of antibodies to *Pf*-IE, *P. falciparum* and *P. vivax* merozoites are shown. Similar patterns were observed with other antigens (data not shown). EGA – estimated gestational age (weeks).
