# Supplementary material for: Maternal-foetal transfer of Plasmodium falciparum and Plasmodium vivax antibodies in a low transmission setting
Source: Sci Rep. 2016 Feb 10;6:20859. doi: 10.1038/srep20859 (PMC4748262; doi:10.1038/srep20859)
Supplement: Supplementary Information [file srep20859-s1.pdf]

Supplementary data

Maternal-foetal transfer of *Plasmodium falciparum* and *Plasmodium vivax* antibodies in a low transmission setting

Sarah C. Charnaud[1], Rose McGready[2,3,4], Asha Herten-Crabb[1,5], Rosanna Powell[1], Andrew Guy[1,6], Christine Langer[1], Jack S. Richards[1,5], Paul R. Gilson[1], Kesinee Chotivanich[7], Takafumi Tsuboi[8], David L. Narum[9], Mupawjay Pimanpanarak[2], Julie A. Simpson[10], James G. Beeson[1,5], François Nosten [2,3,4], Freya J. I. Fowkes[1,10,11,12]

Supplemental Figure 1: Flow chart of women involved in the study

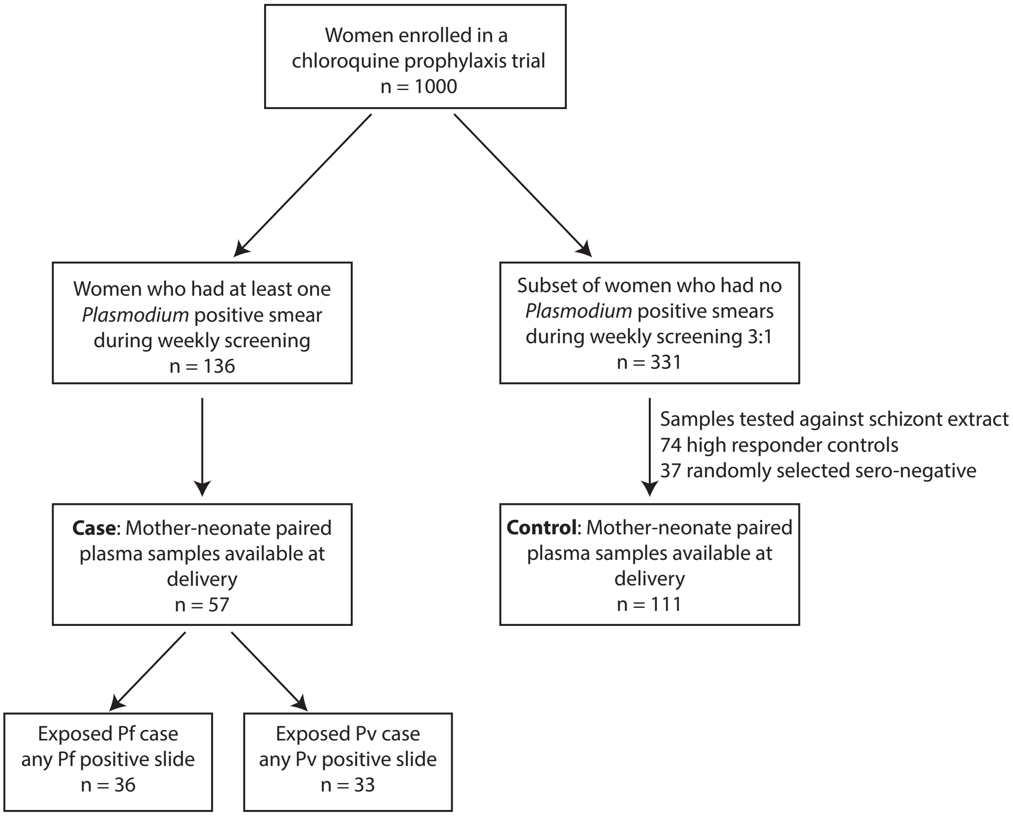

Women were selected from within a placebo controlled trial of chloroquine prophylaxis[1]. Women who had at least one *Plasmodium* positive smear were classified as cases. Within those cases women who had any *P. falciparum* (Pf) positive slide were classified as Pf case, and the same for *P. Vivax* (Pv) cases. If a woman had had both Pf and Pv positive smears during her pregnancy she would be classified as both Pf and Pv case.

Supplemental Table S1: Details of antigens and sera concentrations for high throughput ELISA

Antigen	Coating conc.	Sera conc.	Robotic platform
*Pf*VAR2CSA-DBL5ε [2]	1 µg/mL	1:250	Minitrak [3]
*Pf*DBLα [4]	0.5 µg/mL	1:250	Janus
*Pf*MSP2 [5]	0.5 µg/mL	1:500	Minitrak [3]
*Pf*MSP3 [6]	0.5 µg/mL	1:500	Janus
*Pf*Rh2 [7]	0.5 µg/mL	1:500	Janus
*Pf*AMA1 [8]	0.5 µg/mL	1:2000	Minitrak [3]
*Pf*EBA175 RIII-V [9]	0.5 µg/mL	1:500	Minitrak [3]
*Pf*EBA140 RII [10]	0.5 µg/mL	1:500	Janus
*Pf*EBA140 RIII-V [10]	0.5 µg/mL	1:500	Janus
*Pv*DBP [11]	0.5 µg/mL	1:1000	Janus
*Pv*AMA1 [12]	1 µg/mL	1:250	Minitrak [3]
*Pv*MSP1-19 [13]	0.5 µg/mL	1:1000	Janus
*Pfs*230 [14]	0.5 µg/mL	1:250	Janus
*Pf*CSP [15]	0.5 µg/mL	1:500	Janus
Tetanus toxoid[a]	0.5 µg/mL	1:500	Minitrak [3]
Cytomegalovirus[a]	0.5 µg/mL	1:500	Janus
Measles[a] [3]	1 µg/mL	1:250	Minitrak [3]

High throughput ELISAs performed as per Fowkes et. al 2012[3], except volumes used were adjusted for use on Janus platform. [a]Antigens purchased from PROSPECbio.

Supplemental Table S2. Multivariable linear regression of the effect modification of estimated gestational age on the association between maternal and infant antibody levels

EGA (weeks)	*P. falciparum*						*P. vivax*			
	*Pf*VAR2CSA		*Pf*AMA1		*Pf*EBA175		*Pv*AMA1		*Pv*DBP	
	Coeff. (95%CI)	*P*	Coeff. (95%CI)	*P*	Coeff. (95%CI)	*P*	Coeff. (95%CI)	*P*	Coeff. (95%CI)	*P*
<40	0.70 (0.55-0.84)	Ref.	0.67 (0.49-0.89)	Ref.	0.71 (0.58-0.84)	Ref.	0.59 (0.42-0.75)	Ref.	0.42 (0.28-0.56)	Ref.
40-41	0.15 (-0.04-0.33)	0.11	0.17 (-0.08-0.43)	0.18	0.10 (-0.06-0.27)	0.22	0.16 (-0.06-0.37)	0.15	0.05 (-0.14-0.23)	0.62
>41	0.18 (-0.05-0.41)	0.12	0.21 (-0.07-0.50)	0.15	0.27 (0.08-0.47)	0.007	0.31 (0.03-0.58)	0.028	0.30 (0.04-0.55)	0.019

Multivariable linear regressions were performed on untransformed neonate to maternal antibody ratios. The ratios of antibodies were only calculated in 158 women who were seropositive for each antigen to eliminate very large ratios generated in seronegative women with very low optical densities. Coefficients represent the change in maternal-neonate antibody ratios. Representative examples of antibodies to *Pf*-IE, *P. falciparum* and *P. vivax* merozoites are shown. Similar patterns were observed with all other antigens (data not shown) as a trend of increased ratios towards 1 with increasing EGA. EGA – estimated gestational age (weeks).

References

1. Villegas, L. *et al.* Chloroquine prophylaxis against vivax malaria in pregnancy: a randomized, double-blind, placebo-controlled trial. *Trop. Med. Int. Health* **12,** 209–218 (2007).
2. Avril, M. *et al.* Immunization with VAR2CSA-DBL5 recombinant protein elicits broadly cross-reactive antibodies to placental Plasmodium falciparum-infected erythrocytes. *Infect. Immun.* **78,** 2248–2256 (2010).
3. Fowkes, F. J. I. *et al.* New insights into acquisition, boosting, and longevity of immunity to malaria in pregnant women. *J. Infect. Dis.* **206,** 1612–1621 (2012).
4. Avril, M. *et al.* A restricted subset of var genes mediates adherence of Plasmodium falciparum-infected erythrocytes to brain endothelial cells. *Proceedings of the National Academy of Sciences* **109,** E1782–90 (2012).
5. Stanisic, D. I. *et al.* Immunoglobulin G subclass-specific responses against Plasmodium falciparum merozoite antigens are associated with control of parasitemia and protection from symptomatic illness. *Infect. Immun.* **77,** 1165–1174 (2009).
6. Roussilhon, C. *et al.* Long-term clinical protection from falciparum malaria is strongly associated with IgG3 antibodies to merozoite surface protein 3. *PLoS Med.* **4,** e320 (2007).
7. Reiling, L. *et al.* Evidence that the erythrocyte invasion ligand PfRh2 is a target of protective immunity against Plasmodium falciparum malaria. *J. Immunol.* **185,** 6157–6167 (2010).
8. Hodder, A. N., Crewther, P. E. & Anders, R. F. Specificity of the protective antibody response to apical membrane antigen 1. *Infect. Immun.* **69,** 3286–3294 (2001).
9. Reed, M. B. *et al.* Targeted disruption of an erythrocyte binding antigen in Plasmodium falciparum is associated with a switch toward a sialic acid-independent pathway of invasion. *Proc. Natl. Acad. Sci. U.S.A.* **97,** 7509–7514 (2000).
10. Richards, J. S. *et al.* Identification and prioritization of merozoite antigens as targets of protective human immunity to Plasmodium falciparum malaria for vaccine and biomarker development. *J. Immunol.* **191,** 795–809 (2013).
11. Li, J. & Han, E.-T. Dissection of the Plasmodium vivax reticulocyte binding-like proteins (PvRBPs). *Biochemical and Biophysical Research Communications* **426,** 1–6 (2012).
12. Yildiz Zeyrek, F. *et al.* Serologic markers in relation to parasite exposure history help to estimate transmission dynamics of Plasmodium vivax. *PLoS ONE* **6,** e28126 (2011).
13. Chen, J.-H. *et al.* Measurement of naturally acquired humoral immune responses against the C-terminal region of the Plasmodium vivax MSP1 protein using protein arrays. *Parasitology Research* **109,** 1259–1266 (2011).
14. Tachibana, M. *et al.* N-terminal prodomain of Pfs230 synthesized using a cell-free system is sufficient to induce complement-dependent malaria transmission-blocking activity. *Clin. Vaccine Immunol.* **18,** 1343–1350 (2011).
15. Tsuboi, T. *et al.* Wheat germ cell-free system-based production of malaria proteins for discovery of novel vaccine candidates. *Infect. Immun.* **76,** 1702–1708 (2008).
